# Regulatory Effects of Two Ionic Liquids ([Omim]Br, [Opy]Br) on the Growth and Root Microecology of Maize Seedlings

**DOI:** 10.3390/biology15110839

**Published:** 2026-05-27

**Authors:** Qiuchen Guo, Mengfei Niu, Yiping Wang, Shixu Yang, Qingru Cai, Yulong Ma, Yajun Li, Xiaohong Chen

**Affiliations:** 1 College of Agronomy, Northwest A & F University, Yangling 712100, China; 2024050026@nwafu.edu.cn (Q.G.); yangshixu@nwafu.edu.cn (S.Y.); 15034639628@163.com (Q.C.); 18234425521@163.com (Y.M.); 2 College of Life Sciences, Northwest A & F University, Yangling 712100, China; 15931763331@163.com (M.N.); wangyiping567@163.com (Y.W.)

**Keywords:** ionic liquids, maize, plant growth, rhizosphere microbiome, metabolomics

## Abstract

Accumulation of ionic liquids (ILs) in soil may alter its physicochemical and biological properties. However, the current understanding of their effects on the rhizosphere microenvironment of crop plants remains limited. We examined the effects of two ILs—[Opy]Br and [Omim]Br—which differ in cation structure but share the bromide anion, on maize rhizosphere microbial communities and metabolites at a concentration of 0.6 g/kg soil. Exposure to [Opy]Br and [Omim]Br significantly impaired maize seedling development, with [Opy]Br inducing more severe growth suppression. These phytotoxic effects were also reflected in changes in rhizosphere soil properties. In bacterial communities, [Omim]Br more strongly inhibited membrane transport (e.g., ATP-binding cassette transporters), lipid synthesis, and carbon metabolism, thereby impairing bacterial nutrient uptake and energy metabolism. In fungal communities, saprophytic fungi were activated under both treatments, accelerating organic matter decomposition, whereas pathogens were suppressed, particularly under [Omim]Br treatment. Metabolomic analysis revealed widespread accumulation of amino acids in maize roots following exposure to both ILs, accompanied by significant depletion of the antioxidant glutathione. Carbohydrate metabolism was broadly suppressed, with [Omim]Br exerting a more pronounced inhibitory effect. Hormone levels were generally reduced, with [Opy]Br causing more severe depletion. Overall, both ILs induced oxidative stress, hormonal disruption, and metabolic imbalance in maize. This study provides a reference for evaluating the risks and regulatory potential of ILs in agricultural environments.

## Simple Summary

The growing industrial use of specialized chemicals called ionic liquids raises concerns about their accumulation in farmland and potential harm to crops and soil ecosystems. This study investigated how two common variants affect corn growth, microbes around the roots, and plant chemistry at low, realistic environmental levels. Both substances stunted seedling development and altered soil conditions, with one causing more severe damage. These chemicals disrupted soil bacteria by hindering nutrient absorption and energy production, while simultaneously boosting helpful decomposers and suppressing harmful pathogens. Inside corn roots, the treatments triggered internal cellular stress, depleted natural protective compounds and essential growth hormones, and impaired sugar metabolism. Overall, these industrial liquids create significant physiological strain and metabolic imbalance in corn plants. These findings provide crucial evidence for evaluating the environmental safety of ionic liquids.

## 1. Introduction

Ionic liquids (ILs) are widely used in catalysis, energy, green chemistry, and other fields due to their low volatility, high thermal stability, and tunable properties [1,2]. However, their high chemical stability and persistence may lead to long-term accumulation in the environment, thereby posing ecological risks [3,4]. Most previous studies have primarily focused on the toxicity of ILs in aquatic environments or in crops [5,6,7]. Previous studies indicate that ILs exert toxic effects on enzymes, microorganisms, algae, invertebrates, fish, and plants [8,9,10,11]. Recent research has revealed that the toxicity of ILs to plants is closely related to cation structure and alkyl chain length [12]. For instance, tetrabutylammonium chloride-based ILs significantly inhibit root growth in wheat and cucumber [13]. The toxicity of pyridinium bromides ([C3py]Br and [C5py]Br) to rapeseed seedlings increases with increasing side-chain length [14]. The soil environment is fundamental to crop growth. When ILs accumulate in soil, they may affect soil biodiversity by inducing biochemical and biophysical changes, which in turn influence plant growth [15,16,17,18]. Research on herbicide-based ILs indicates that quaternary ammonium cations in glyphosate-based ILs inhibit mineralization through soil adsorption. This adsorption property may pose environmental risks [19]. Other studies have found that the toxic effects of choline-based ionic liquids ([Chol][DHP]) on soil microorganisms are significantly influenced by soil organic matter content, making soil organic matter abundance a key factor in assessing their toxicity [20].

The rhizosphere is a specialized zone formed through plant–soil–microbial interactions, and rhizosphere soil is closely linked to plant growth and development [21,22]. Currently, research on ILs in rhizosphere soil remains limited. Soil microorganisms are key participants in numerous soil chemical processes, such as organic matter mineralization and nutrient cycling [23]. Some studies indicate that alkyl chain length is a key factor influencing IL toxicity, with long-chain ILs (e.g., [C10mim]Br) causing the most severe disruption to soil microorganisms and nitrogen cycling [24]. In addition, hydrophobic cations in ILs can significantly alter rhizosphere soil microbial community structure [25]. This is consistent with our current research results. Imidazolium and pyridinium ionic liquids also pose risks to soil ecology. Studies have also indicated that ILs may cause abnormal increases in gene abundance and microbial diversity in soil. Certain HIL-treated agricultural soils showed elevated abundances of soxA and phnJ genes, although this effect proved transient [19]. ILs can modulate the physicochemical properties of compost, promote lignocellulose degradation, optimize microbial community structure, and stimulate the growth of Actinomycetes and Proteobacteria [26]. Research has indicated that [Bmim]Cl significantly reduces the abundance of acidifying bacteria and methanogens, thereby affecting microbial community stability [27]. Ecological risks may be mitigated by using dimethyl-substituted variants or through anion optimization [28]. However, these studies have not addressed the interactions among soil physicochemical properties, microbial communities, and rhizosphere metabolism.

Existing evidence suggests that ILs in soil influence the relative abundance, community structure, and functional profiles of the plant rhizosphere microbiome. Furthermore, ILs with different compositions and structures may exert distinct effects on the rhizosphere microenvironment and root metabolites. Pyridinium-based and imidazolium-based ionic liquids are two of the most typical nitrogen-containing aromatic ring cation ionic liquids (structures are shown in Figure S1). The research on imidazolium-based ionic liquids is relatively thorough, and the ecological risk model is relatively mature; however, the overall toxicological database of pyridinium-based ionic liquids is not complete. These two ionic liquids were selected to compare the differences in ecological toxicity to corn caused by ionic liquids with different aromatic ring structures. This study selected two structurally distinct ILs—[Omim]Br and [Opy]Br—to compare their effects on the maize rhizosphere. Their impacts on the physicochemical properties, microbial community composition, and metabolic profile of rhizosphere soil were subsequently analyzed. The toxicological impacts of [Omim]Br and [Opy]Br on maize seedling rhizosphere soil were systematically evaluated, with particular emphasis on cation-specific effects. Multidimensional analysis—encompassing microbial community dynamics, metabolic profiles, and root morphological responses—was employed to elucidate the structure–toxicity relationships governing rhizosphere perturbation. Additionally, functional prediction tools, including PICRUSt2 and FUNGuild, were applied to infer shifts in the functional potential of maize rhizosphere microbial communities. These analyses, combined with root metabolite profiling, provide insights into how ILs influence root-zone microbiomes and highlight associated ecological risks in soil systems.

## 2. Materials and Methods

### 2.1. Chemicals

The ILs 1-octyl-3-methylimidazolium bromide ([Omim]Br) and N-octylpyridinium bromide ([Opy]Br) (both 99% purity) were purchased from the Lanzhou Institute of Chemical Physics, Chinese Academy of Sciences, and used as received. Figure S1 shows the molecular structures of these two ILs.

All other reagents were of analytical grade.

### 2.2. Experimental Design and Sampling

Maize seeds (Zhengdan 958, *Zea mays* L.) used in this study were purchased from Doneed Seed Company in Beijing, China. The seeds were surface-sterilized with 75% ethanol for 3 min, rinsed three times with distilled water, soaked for 12 h at room temperature, and germinated in the dark at 28 °C for 48 h. Uniformly germinated seeds were transferred to plastic trays for further cultivation.

Soil was collected from corn fields at the Northwest A&F University Experimental Station and air-dried under natural conditions. Soil samples (the basic physicochemical properties are shown in Table 1) were sieved through a 20-mesh screen and then mixed with vermiculite at a 3:1 ratio (soil:vermiculite). The mixture was divided into three portions: one treated with water (control), one with 1-octyl-3-methylimidazolium bromide, and one with N-octylpyridinium bromide. After thorough mixing, an IL concentration of 0.6 g/kg soil was achieved. Uniformly germinated maize seeds were sown in 800 g of potting soil, covered with an additional 200 g of soil, and irrigated to a final pot weight of 1200 g. Pots were watered regularly to maintain a total weight of 1200 ± 50 g, and the temperature was maintained at 25 ± 1 °C. After 30 days of cultivation, rhizosphere soil was collected, and its physicochemical and biological properties were determined.

### 2.3. Soil Physicochemical Properties Analysis

Rhizosphere soil refers to the soil layer in direct contact with plant roots (typically within a few millimeters of the root surface) and exhibits significant differences from non-rhizosphere soil. During sample collection, rhizosphere and non-rhizosphere soils were carefully distinguished to prevent cross-contamination. After 30 days of treatment, rhizosphere soil was collected from each maize treatment group. When harvesting maize plants, loosely attached soil (non-rhizosphere soil) was gently shaken from the roots, while soil tightly adhering to the root surface (rhizosphere soil) was retained [29]. Fresh soil samples were sieved and stored at −80 °C for subsequent physicochemical and metabolomic analyses. Each treatment comprised four biological replicates obtained from distinct pots.

Soil physicochemical properties—including pH, soil water content, total nitrogen (TN), soil organic carbon, ammonium nitrogen (NH_4_^+^–N), nitrate nitrogen (NO_3_^−^–N), available phosphorus (AP), and available potassium (AK)—were determined using a pH meter, potassium dichromate oxidation, Kjeldahl digestion, alkali diffusion, and standard colorimetric methods. Detailed descriptions of the analytical procedures are provided in our previous studies [30].

### 2.4. Microbial Community Composition and Functional Analysis

Samples were sent to Major Biotechnology (Shanghai, China) for interactive cloud-based environmental microbial diversity analysis. High-throughput sequencing technology was used to systematically analyze maize rhizosphere soil microbial communities. The 16S rRNA gene was sequenced to characterize bacterial and archaeal community structure and diversity, whereas fungal internal transcribed spacer (ITS) sequencing, targeting the ITS1 and ITS2 regions, enabled high-resolution taxonomic identification and functional characterization of fungal communities.

Functional potential was predicted for bacteria and fungi using PICRUSt2 and FUNGuild. PICRUSt2 inferred bacterial metabolic pathways (e.g., carbon and nitrogen cycling, stress responses) by mapping 16S rRNA gene data to the Kyoto Encyclopedia of Genes and Genomes (KEGG) database, whereas FUNGuild classified fungal ecological guilds based on ITS-derived taxonomic assignments [31,32].

### 2.5. Analysis of Rhizosphere Metabolite Differences and Functional Pathways

Metabolite extraction and detection methods:

Freeze-dried rhizosphere soil (25 mg) was extracted with 500 μL of pre-chilled methanol:water (3:1, *v*/*v*) through three cycles of homogenization (40 Hz, 4 min) followed by ice-bath sonication (5 min). Following overnight incubation at 4 °C, samples were centrifuged at 13,800 × *g* for 15 min. The supernatant was filtered through a 0.22 μm membrane and diluted fivefold. Quality control samples were prepared by pooling equal aliquots (50 μL) from each extract. Chromatographic separation was performed on an EXION LC system (SCIEX) equipped with an ACQUITY HSS T3 column (100 mm × 2.1 mm i.d., 1.8 μm; Waters, Milford, MA, USA), using 0.1% formic acid in water (A) and acetonitrile (B) as the mobile phases. The column and autosampler temperatures were maintained at 40 °C and 4 °C, respectively, with an injection volume of 2 μL. Data acquisition and processing were conducted using SCIEX Analyst Workstation Software (v1.6.3).

Data Analysis Methods:

Data were processed using Excel 2019 and R software (Version1.6.2) [30]. Statistical significance (*p* < 0.05) was assessed by one-way analysis of variance between the [Omim]Br and [Opy]Br groups. Significantly different metabolites were identified based on variable importance in projection values > 1.0 and *p* < 0.05.

## 3. Results and Discussion

### 3.1. Effects of Two ILs on Early Maize Growth

**Figure 1:**
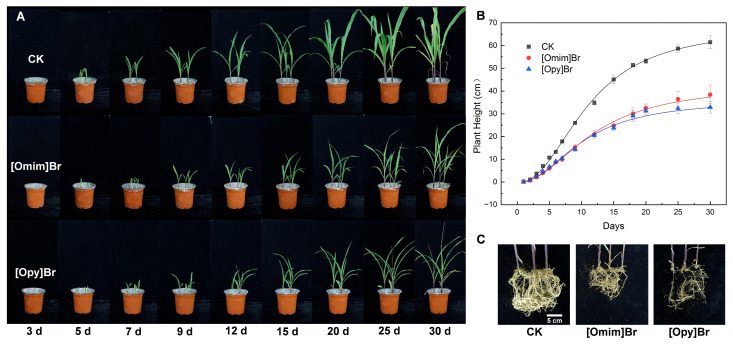
Morphology of maize seedlings (**A**) and roots (**C**) subjected to two ionic liquid treatments; and (**B**) represents the plant height of corn seedlings over 30 days of two treatments.

Following 30 days of ILs exposure, maize seedling growth and development were assessed using standardized morphological and physiological measurements. Morphological changes in maize seedlings over the 30-day period are shown in Figure 1. Experimental analysis revealed that both ILs significantly inhibited the aboveground and belowground growth of maize seedlings. Despite their differing cationic structures, [Omim]Br and [Opy]Br share identical alkyl chain lengths, resulting in comparable suppression of shoot biomass. In contrast, root architecture was differentially affected: [Opy]Br elicited more severe inhibition, significantly reducing lateral root formation and root hair proliferation. This heightened sensitivity of roots may stem from their direct exposure to ILs in the rhizosphere, rendering them more vulnerable than aboveground tissues to chemical stress [33].

These observations indicate that IL toxicity exhibits a dose-dependent relationship, with increasing concentrations leading to enhanced phytotoxic or ecotoxic effects. This correlation is particularly pronounced at high concentrations, whereas at low concentrations, ILs may behave similarly to inorganic salts or plant growth hormones, thereby promoting plant growth and development [34,35,36].

Figure 1B illustrates the treatment-specific effects of ILs on maize shoot elongation. Notably, [Opy]Br induced severe growth retardation, with significant suppression of plant height evident by day 10. Thereafter, height increments ceased, plateauing from day 20 onward—a pattern indicative of irreversible growth arrest and likely reflecting the compound’s strong phytotoxicity under prolonged exposure. The [Omim]Br treatment also suppressed maize plant height, albeit to a lesser extent than [Opy]Br; however, plant height remained significantly lower than that of the control group (CK).

### 3.2. Analysis of Maize Rhizosphere Soil Properties

Both IL treatments significantly reduced rhizosphere TN content relative to the control, with the [Opy]Br treatment inducing a more pronounced reduction. This nitrogen decline parallels findings in metal-polluted soils, where heavy metal stress impairs microbial nitrogen cycling and reduces bioavailable nitrogen pools. Such effects may result from heavy metal toxicity to soil microorganisms, which compromises nitrogen cycling efficiency by damaging microbial cell membranes or inhibiting key enzymes, such as nitrifying enzymes [37], thereby reducing TN content. These findings are consistent with our results and suggest that ILs may exert toxicity analogous to that of heavy metals in soil microbial communities, thereby disrupting nitrogen cycling. The more pronounced nitrogen depletion observed in the [Opy]Br group may be attributed to cation-specific properties, such as enhanced membrane permeability or stronger binding affinity to microbial enzymes [38].

Ammonium nitrogen content was significantly higher in the [Omim]Br treatment than in the other treatments. This increase likely resulted from inhibition of root nitrogen uptake and suppression of key nitrogen-cycling microorganisms in the soil [39], leading to abnormal accumulation of ammonium nitrogen.

**Figure 2:**
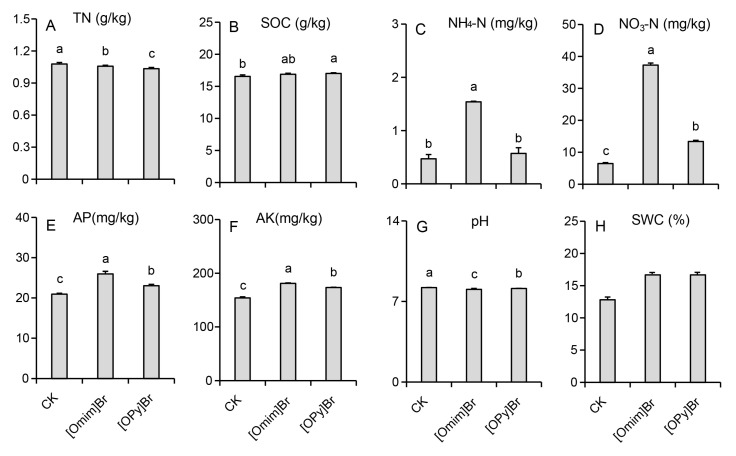
Effects of two ionic liquids on the physicochemical properties ((**A**)–(**H**) each represent TN, SOC, NH_4_-N, NO_3_-N, AK, AP, pH, SWC) of maize rhizosphere soil (a–c represents the significant difference).

Figure 2 shows that both IL treatments significantly increased the AP and AK contents in the soil. Under normal conditions, plants absorb AP and AK from the soil, causing nutrient concentrations in the rhizosphere to gradually decrease with increasing distance from the root surface and forming nutrient depletion zones. However, when ILs exert toxic effects on roots, their capacity to absorb mineral nutrients declines. As a result, readily AP and AK are not effectively taken up by roots, leading to their accumulation in the rhizosphere and elevated concentrations of these nutrients [40,41].

Soil moisture content also significantly increased in both treatment groups. Under normal conditions, roots maintain a dynamic equilibrium of soil moisture by absorbing water from the surrounding soil. When root water uptake capacity declines, soil moisture is not effectively absorbed, leading to increased soil moisture content. Organic matter content significantly increased in the [Opy]Br group, potentially due to the inhibitory effect of the IL on microbial decomposition of organic matter. The reduced decomposition rate resulted in organic matter accumulation [38].

In conclusion, the cations in [Opy]Br and [Omim]Br may negatively affect maize growth by altering soil physicochemical properties, influencing rhizosphere microbial activity, or regulating physiological and metabolic processes in the maize rhizosphere [30,42,43].

### 3.3. Analysis of Microbial Characteristics in Maize Rhizosphere Soil

#### 3.3.1. Analysis of Bacterial Community Composition and Relative Abundance

**Figure 3:**
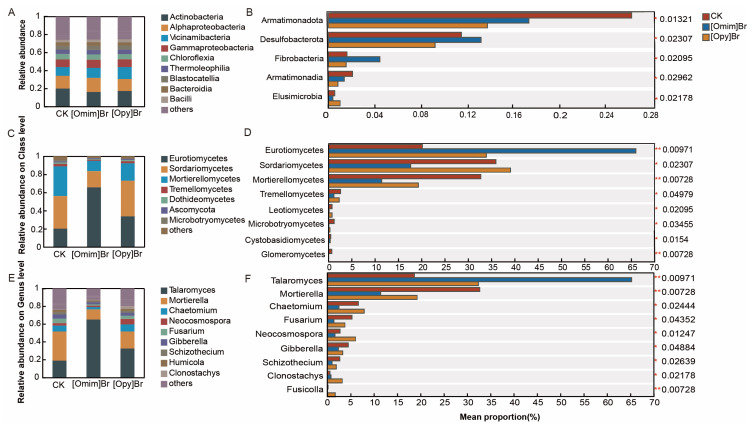
Bacterial and fungal community composition in maize rhizosphere soil under different treatments. Relative abundance profiles (**A**,**C**,**E**) and significance analyses (**B**,**D**,**F**) are shown for bacteria at the class level (**A**,**B**) and fungi at the class (**C**,**D**) and phylum levels (**E**,**F**). * represent significant differences, ** indicating significant differences.

As the core interface of plant–soil interactions, the rhizosphere microbiome (encompassing bacteria, fungi, and archaea) plays a critical role in regulating crop growth through mechanisms such as nutrient cycling, soil structure improvement, and pathogen antagonism [44,45,46,47,48]. The bacterial community structure at the class level is illustrated in Figure 3A. The bacterial classes with relative abundances exceeding 5% across all three treatment groups, ranked in descending order, were Actinobacteria, Alphaproteobacteria, Vicinamibacteria, Gammaproteobacteria, and Chloroflexi. Actinobacteria, Alphaproteobacteria, and Vicinamibacteria constituted the dominant bacterial classes, with their combined relative abundance exceeding 40% in all three treatment groups. Compared with the 20.1% abundance in the control group, the relative abundance of Actinobacteria decreased to 16.1% in the [Omim]Br group and 17.2% in the [Opy]Br group. The relative abundance of Alphaproteobacteria increased from 14.1% in the control to 15.7% in the [Omim]Br treatment but decreased to 13.4% in the [Opy]Br group. Vicinamibacteria abundance increased in both IL treatments, rising from 9.5% in the control to 11.0% in the [Omim]Br group and 13.3% in the [Opy]Br group. In contrast, no statistically significant changes were observed in Gammaproteobacteria or Chloroflexi across treatments.

**Figure 4:**
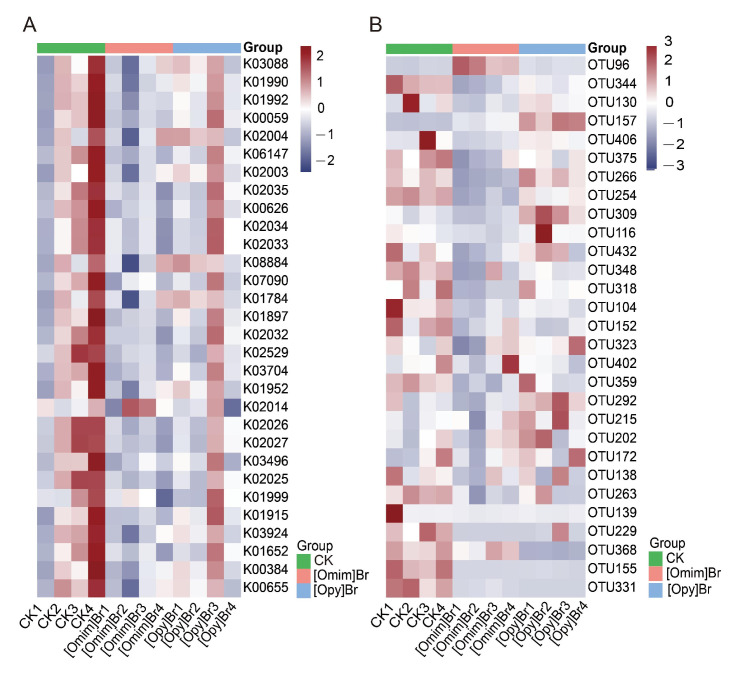
PICRUSt2-predicted functional pathways of bacterial communities (**A**) and FUNGuild-based functional group analysis of fungal communities (**B**) in rhizosphere soil under three treatment conditions.

As shown in Figure 4A, the top 30 functional pathways exhibited varying degrees of downregulation in both treatment groups, with the membrane transport pathway showing the most pronounced decrease. This category includes ATP-binding cassette (ABC) transporter families involved in substance transport across membranes, such as K01990, K01992, K06147, and K02003, suggesting that IL stress may impair bacterial transmembrane transport functions. Additionally, K00059, K01897, and K00655, which are associated with fatty acid and phospholipid biosynthesis, were also significantly downregulated, indicating that ILs suppress lipid metabolic activity in bacterial communities. The downregulation of carbohydrate metabolism (K00626) and amino acid metabolism (K01915) may reflect inhibition of basal microbial metabolic activities by ILs [49,50,51]. Overall, the [Omim]Br treatment group exhibited more pronounced downregulation across multiple functional modules, including membrane transport, carbohydrate metabolism, and genetic information processing. In contrast, the inhibitory effects of the [Opy]Br treatment were relatively moderate, suggesting that [Omim]Br may exert a stronger suppressive or disruptive influence on bacterial physiological activities. Specifically, within the membrane transport system, key genes encoding ABC transporters (K06147), peptide/nickel transport systems (K02035, K02033, K02032), and polysaccharide transport systems (K02026, K02027, K02025) exhibited greater downregulation in the [Omim]Br group (average reduction of 15–23%) than in the [Opy]Br group (7–19%). These findings suggest that [Omim]Br more effectively impairs bacterial nutrient uptake—including peptides, nickel ions, and polysaccharides—thereby compromising metabolic activity. In terms of metabolic regulation, key enzymes involved in ketone body and cholesterol synthesis (acetoacetyl-CoA thiolase, K00626) and phospholipid biosynthesis (K00655) exhibited greater downregulation in the [Omim]Br group than in the [Opy]Br group. This finding suggests that [Omim]Br more substantially disrupts bacterial lipid and energy metabolism. Furthermore, the lactose operon repressor (K02529), a key regulator in genetic information processing, was downregulated by 20.4% in the [Omim]Br group and 19.6% in the [Opy]Br group. These results indicate that both treatments strongly suppress transcriptional regulation of genes associated with carbohydrate metabolism.

#### 3.3.2. Analysis of Soil Fungal Community Composition and Relative Abundance

The fungal community structure at the class level, as depicted in Figure 3C, was characterized by the consistent dominance of three major classes: *Eurotiomycetes*, *Sordariomycetes*, and *Mortierellomycetes*. Collectively, these three classes totally accounted for 88.8%, 94.9%, and 92.1% of the total fungal community in the control, [Omim]Br, and [Opy]Br treatment groups. *Eurotiomycetes* and *Mortierellomycetes* exhibited highly significant differences among treatments. Compared with the control group, both experimental groups showed an increased relative abundance of *Eurotiomycetes* and a decreased relative abundance of *Mortierellomycetes*. At the genus level (Figure 3E), the top five fungal genera by relative abundance in the control group were *Mortierella*, *Talaromyces*, *Chaetomium*, *Neocosmospora*, and *Fusarium*. In the [Omim]Br group, the dominant genera were *Talaromyces, Mortierella*, unclassified *Ascomycota*, *Chaetomium*, and *Schizothecium*, whereas in the [Opy]Br group, they were *Talaromyces*, *Mortierella*, unclassified *Ascomycota*, *Schizothecium*, and *Humicola*. Notably, *Talaromyces* consistently dominated across all groups, accounting for 18.7% (control), 64.9% ([Omim]Br), and 32.2% ([Opy]Br) of the total fungal relative abundance; *Mortierella* was the second most abundant, occupying the three groups for 32.7% (control), 11.5% ([Omim]Br), and 19.2% ([Opy]Br). Statistical analysis of intergroup differences revealed highly significant differences in the relative abundances of *Talaromyces* and *Mortierella*. Specifically, *Mortierella* showed a significant increase in the [Omim]Br treatment group, whereas *Talaromyces* exhibited significant decreases in both the [Omim]Br and [Opy]Br treatment groups. No major differences were observed among the remaining genera.

Saprophytic and degradative fungal OTUs 96 and 157 exhibited significant increases in relative abundance under [Omim]Br and [Opy]Br treatments, with fold changes of 18.74 and 29.97, respectively, suggesting strong stimulation by both ILs. Functionally, these fungi play a critical role in degrading recalcitrant organic matter—particularly lignocellulose and chitin—thereby contributing to carbon cycling and soil organic matter dynamics. Concurrently, OTU172, a putative nitrogen-cycling fungus, exhibited moderate enrichment under the [Opy]Br treatment but was suppressed under [Omim]Br. This differential response suggests that [Opy]Br may facilitate soil ammonia oxidation or nitrification processes, potentially enhancing soil nitrogen availability [52,53]. In contrast, the relative abundance of most plant pathogenic fungi (e.g., OTU344, OTU406, OTU432) significantly decreased under both [Omim]Br and [Opy]Br treatments. The key difference between the two treatments is that [Omim]Br exerted stronger suppression across multiple plant pathogenic fungal groups, whereas [Opy]Br showed comparatively milder inhibition. For instance, OTU344 decreased by 68% and 36% under [Omim]Br and [Opy]Br treatments, respectively, whereas OTU406 decreased by 78% and 45%, respectively. Furthermore, certain symbiotic biotrophic fungi (e.g., OTU309, OTU292) exhibited increased abundance under the [Opy]Br treatment but decreased abundance under the [Omim]Br treatment. The [Opy]Br treatment may promote the secretion of plant hormones or antimicrobial compounds by these fungi, thereby indirectly inhibiting pathogens.

### 3.4. Metabolomics Analysis of Maize Rhizosphere Soil

Metabolomic profiling of maize seedling roots using non-targeted LC–MS/MS identified 357 annotated metabolites across the three treatment groups. Multivariate statistical analysis based on orthogonal partial least squares discriminant analysis identified 196 metabolites that exhibited significant differential accumulation. The [Omim]Br treatment resulted in 113 upregulated and 83 downregulated metabolites, whereas the [Opy]Br treatment resulted in 110 upregulated and 86 downregulated metabolites.

**Figure 5:**
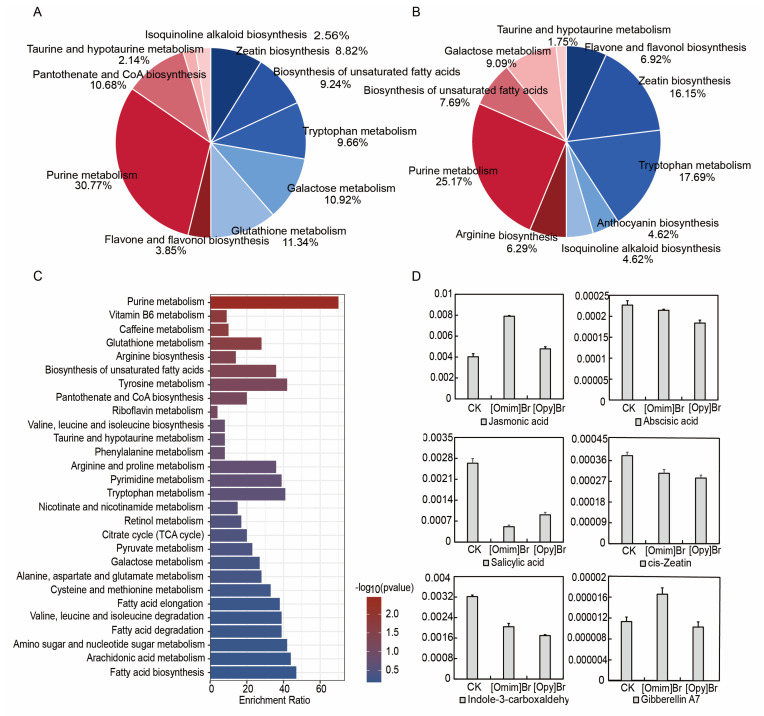
Significantly upregulated and downregulated metabolite categories in [Omim]Br-treated (**A**) and [Opy]Br-treated (**B**) plants compared with CK plants. Panel (**C**) shows significantly enriched metabolic pathways under different treatments, and panel (**D**) shows changes in plant hormone levels across treatments.

Metabolomic analysis revealed widespread accumulation of amino acid–related metabolites in both IL treatment groups, with phenylalanine, tryptophan, glutamine, and leucine showing significantly elevated abundances. In contrast, glutathione was markedly depleted, decreasing by 34.1% under [Omim]Br and 34.8% under [Opy]Br relative to the control. This depletion may indicate redox imbalance and impaired detoxification capacity in maize roots under IL stress, reflecting oxidative stress induced by reactive oxygen species accumulation, which exacerbates membrane phospholipid damage [38,54,55]. Meanwhile, metabolomic profiling showed that 1-O-caffeoylglucose and but-3-enyl glucuronic acid thiol were significantly downregulated under both [Omim]Br and [Opy]Br treatments, with the [Omim]Br group exhibiting a greater magnitude of reduction. Fatty acid metabolites exhibited distinct accumulation patterns across the two treatments, potentially resulting from enhanced membrane phospholipid degradation under IL exposure, leading to compromised cell membrane integrity [5,6,7,49]. Changes in plant hormones across the three treatment groups are shown in Figure 5D. Hormonal profiling revealed significant reductions in key phytohormones under both IL treatments. In the [Omim]Br group, ABA, I3C, IAA, and CZ levels declined by 5.4%, 36.5%, 10.6%, and 20.0%, respectively. In the [Opy]Br group, greater suppression was observed for ABA (−18.9%), GA7 (−9.2%), I3C (−47.2%), IAA (−41.3%), and CZ (−25.5%). Notably, the IAA/CZ ratio decreased from 8.4 (control) to 6.69 ([Omim]Br) and 5.93 ([Opy]Br), implying a relative shift toward cytokinin-mediated growth suppression under IL exposure. Similarly, the ABA/JA ratio decreased from 0.056 (control) to 0.027 ([Omim]Br) and 0.038 ([Opy]Br). These results indicate that both IL treatments weakened auxin–cytokinin crosstalk and enhanced antagonistic interactions between ABA and JA, potentially leading to root growth inhibition and arrest of lateral root development [56,57,58].

Metabolic pathway enrichment analysis of the 196 differentially accumulated metabolites was conducted using the KEGG database (Figure 5C). These metabolites were significantly enriched in pathways including purine metabolism, vitamin B6 metabolism, caffeine metabolism, glutathione metabolism, and arginine biosynthesis. Separate enrichment analyses were performed for the two IL treatment groups (Figure 5A,B). Metabolic pathways significantly upregulated in the [Omim]Br-treated group were ranked by statistical significance (from highest to lowest). The top-ranking pathways included flavone and flavonol biosynthesis, purine metabolism, pantothenate and CoA biosynthesis, taurine and hypotaurine metabolism, and isoquinoline alkaloid biosynthesis. The most significantly downregulated pathways, ranked in descending order of statistical significance, were zeatin biosynthesis, biosynthesis of unsaturated fatty acids, tryptophan metabolism, galactose metabolism, and glutathione metabolism. Metabolic pathways significantly upregulated in the [Opy]Br-treated group were also ranked by statistical significance (descending order). The top-ranking pathways included arginine biosynthesis, purine metabolism, biosynthesis of unsaturated fatty acids, galactose metabolism, and taurine and hypotaurine metabolism. The most significantly downregulated pathways, ranked in descending order of statistical significance, were flavone and flavonol biosynthesis, zeatin biosynthesis, tryptophan metabolism, anthocyanin biosynthesis, and isoquinoline alkaloid biosynthesis. The patterns of upregulation and downregulation differed between the two treatment groups. For instance, flavone and flavonol biosynthesis was upregulated in the [Omim]Br group but downregulated in the [Opy]Br group, whereas galactose metabolism was downregulated in the [Omim]Br group but upregulated in the [Opy]Br group. These findings suggest that structurally distinct ILs differentially modulate maize rhizosphere metabolism, highlighting the need for further experimental validation.

## 4. Conclusions

[Omim]Br and [Opy]Br significantly modulated rhizosphere microbial community structure and seedling metabolic profiles, indicating a dual impact on plant–microbe interactions.

### 4.1. Soil

IL treatments altered the physicochemical properties of rhizosphere soil, reducing TN content while increasing organic matter and AP and AK levels.

### 4.2. Bacteria

Compared with [Opy]Br, [Omim]Br exhibited stronger inhibitory effects across multiple core functional pathways, particularly membrane transport and metabolic regulation, suggesting that it may achieve more effective physiological suppression by broadly disrupting bacterial material transport and energy metabolism.

### 4.3. Fungi

Different IL treatments significantly altered the functional structure of soil fungal communities. Both [Omim]Br and [Opy]Br treatments strongly activated saprophytic decomposers (OTU96 and OTU157), potentially releasing nutrients in the short term but intensifying carbon mineralization in the long term. Both treatments significantly reduced the abundance of plant pathogens, with [Omim]Br exhibiting stronger inhibitory effects.

### 4.4. Metabolites

Under both treatments, amino acids (e.g., phenylalanine and tryptophan) accumulated in maize roots, whereas the antioxidant glutathione decreased significantly, potentially inducing oxidative stress through reactive oxygen species accumulation and consequent cell membrane damage. Both [Omim]Br and [Opy]Br inhibited hormone levels, reduced carbohydrate metabolism, and disrupted redox balance. However, [Omim]Br more substantially downregulated carbohydrate metabolites, whereas [Opy]Br more strongly depleted hormones and upregulated galactose metabolism. These findings indicate distinct targets and regulatory directions underlying their inhibitory effects on maize rhizosphere metabolism.

## Figures and Tables

**Table 1:** Physicochemical properties of the soil.

Parameter	Value
pH	6.43
Organic matter (%)	2.04
Total nitrogen (g kg^−1^)	1.72
Total phosphorus (g kg^−1^)	1.14
Cation exchange capacity (cmol kg^−1^)	25.4
Clay content (%)	50.6
Sand content (%)	9.0

## Data Availability

The raw data supporting the conclusions of this article will be made available by the authors on request.

## Supplementary Materials

The following supporting information can be downloaded at: https://www.mdpi.com/article/10.3390/biology15110839/s1, Figure S1: The molecular structure of the assessed two ionic liquids; Table S1: List of acronyms.
