# Supplementary material for: Regulatory Effects of Two Ionic Liquids ([Omim]Br, [Opy]Br) on the Growth and Root Microecology of Maize Seedlings
Source: Biology (Basel). 2026 May 27;15(11):839. doi: 10.3390/biology15110839 (PMC13255988; doi:10.3390/biology15110839)
Supplement: Supplementary file 1 [file biology-15-00839-s001.zip › biology-4303221-supplementary.pdf]

Supplementary Materials

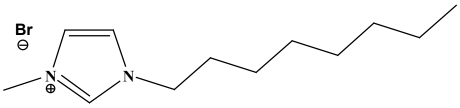

1-Octyl-3-methylimidazolium bromide

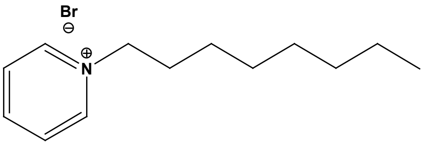

N-octyl pyridinium bromide

Figure S1. The molecular structure of the assessed two ionic liquids

Table S1.List of acronyms

**English abbreviation**	**English full name**
ILs	Ionic Liquids
[Omim]Br	1-octyl-3-methylimidazolium bromide
[Opy]Br	N-octylpyridinium bromide
TN	Total Nitrogen
SOC	Soil Organic Carbon
NH_4-N	Ammonium Nitrogen
NO_3-N	Nitrate Nitrogen
AK	Available Potassium
AP	Available Phosphorus
pH	potential of Hydrogen
SWC	Soil Water Content
